# The impact of histopathology and *NAB2*–*STAT6* fusion subtype in classification and grading of meningeal solitary fibrous tumor/hemangiopericytoma

**DOI:** 10.1007/s00401-018-1952-6

**Published:** 2018-12-24

**Authors:** Karen Fritchie, Kassandra Jensch, Evgeny A. Moskalev, Alissa Caron, Sarah Jenkins, Michael Link, Paul D. Brown, Fausto J. Rodriguez, Andrew Guajardo, Daniel Brat, José E. Velázquez Vega, Arie Perry, Ashley Wu, David R. Raleigh, Sandro Santagata, David N. Louis, Priscilla K. Brastianos, Alexander Kaplan, Brian M. Alexander, Sabrina Rossi, Fabio Ferrarese, Florian Haller, Caterina Giannini

**Affiliations:** 1 Anatomic Pathology, Department of Laboratory Medicine and Pathology, Mayo Clinic, 200 First Street, SW, Rochester, MN 55905 USA; 2 Institute of Pathology, University Hospital Erlangen, Erlangen, Germany; 3 Department of Health Sciences Research, Mayo Clinic, Rochester, MN USA; 4 Department of Neurosurgery, Mayo Clinic, Rochester, MN USA; 5 Department of Radiation Oncology, Mayo Clinic, Rochester, MN USA; 6 Department of Pathology, Johns Hopkins, Baltimore, MD USA; 7 Department of Pathology, Northwestern University Feinberg School of Medicine, Chicago, IL USA; 8 Department of Pathology, Children’s Healthcare of Atlanta, Atlanta, GA USA; 9 Department of Pathology, University of California, San Francisco, CA USA; 10 Department of Radiation Oncology, University of California, San Francisco, CA USA; 11 Department of Neurological Surgery, University of California, San Francisco, CA USA; 12 Department of Pathology, Brigham and Women’s Hospital, Boston, MA USA; 13 Department of Pathology, Massachusetts General Hospital and Harvard Medical School, Boston, MA USA; 14 Department of Hematology/Oncology, Massachusetts General Hospital and Harvard Medical School, Boston, MA USA; 15 Department of Neuro-oncology, Massachusetts General Hospital and Harvard Medical School, Boston, MA USA; 16 Department of Radiation Oncology, Dana-Farber Cancer Institute, Boston, MA USA; 17 Department of Pathology and Molecular Genetics, Ospedale Ca’Foncello, Treviso, Italy; 18 Department of Radiation Oncology, Ospedale Ca’Foncello, Treviso, Italy

**Keywords:** Meningeal hemangiopericytoma, Meningeal solitary fibrous tumor, *NAB2*–*STAT6*, STAT6

## Abstract

Meningeal solitary fibrous tumor (SFT)/hemangiopericytoma (HPC) is a rare tumor with propensity for recurrence and metastasis. Although multiple classification schemes have been proposed, optimal risk stratification remains unclear, and the prognostic impact of fusion status is uncertain. We compared the 2016 WHO CNS tumor grading scheme (CNS-G), a three-tier system based on histopathologic phenotype and mitotic count, to the 2013 WHO soft-tissue counterpart (ST-G), a two-tier system based on mitotic count alone, in a cohort of 133 patients [59 female, 74 male; mean age 54 years (range 20–87)] with meningeal SFT/HPC. Tumors were pathologically confirmed through review of the first tumor resection (*n* = 97), local recurrence (*n* = 35), or distant metastasis (*n* = 1). A STAT6 immunostain showed nuclear expression in 132 cases. *NAB2*–*STAT6* fusion was detected in 99 of 111 successfully tested tumors (89%) including the single STAT6 immunonegative tumor. Tumors were classified by CNS-G as grade 1 (*n* = 43), 2 (*n* = 41), or 3 (*n* = 49), and by ST-G as SFT (*n* = 84) or malignant SFT (*n* = 49). Necrosis was present in 16 cases (12%). On follow-up, 42 patients had at least one subsequent recurrence or metastasis (7 metastasis only, 33 recurrence only, 2 patients had both). Twenty-nine patients died. On univariate analysis, necrosis (*p* = 0.002), CNS-G (*p* = 0.01), and ST-G (*p* = 0.004) were associated with recurrence-free (RFS) but not overall survival (OS). *NAB2*–*STAT6* fusion type was not significantly associated with RFS or OS, but was associated with phenotype. A modified ST-G incorporating necrosis showed higher correlation with RFS (*p* = 0.0006) and remained significant (*p* = 0.02) when considering only the primary tumors. From our data, mitotic rate and necrosis appear to stratify this family of tumors most accurately and could be incorporated in a future grading scheme.

**Electronic supplementary material:**

The online version of this article (10.1007/s00401-018-1952-6) contains supplementary material, which is available to authorized users.

## Introduction

Meningeal solitary fibrous tumor (SFT)/hemangiopericytoma (HPC) is an often aggressive mesenchymal tumor of fibroblastic origin that arises from the cranial or spinal dura [14]. Although SFT and HPC were initially thought to represent distinct entities, the identification of *NAB2*–*STAT6* fusion as a defining molecular alteration in both tumors has led to the unification of these entities at both dural and extra-dural sites. While the *NAB2*–*STAT6* fusion seems to be unique to SFT/HPC, its detection may be difficult unless whole-genome sequencing is applied to detect breakpoints that occur both in exon and intron boundaries [5, 16]. STAT6 nuclear expression is accepted as a sensitive surrogate of all fusions, which causes consistent nuclear relocation of STAT6 [9, 14, 17]. Tumors arising from the meninges tend to exhibit high rates of local recurrence with propensity for metastasis outside the central nervous system (CNS), but prognostication on histopathology alone has been notoriously difficult regardless of site of origin. The current 2016 WHO CNS grading scheme incorporates phenotype and mitotic rate to stratify tumors into three groups (grade 1, 2, and 3), while non-meningeal soft-tissue tumors are classified by mitotic rate alone into SFT and malignant SFT according to the 2013 WHO classification for soft-tissue tumors [10, 14]. Our earlier work suggested that tumors harboring the *NAB2* exon 4–*STAT6* exon 3 fusion variant exhibited morphologic features similar to the conventional solitary fibrous tumor, while there was a trend toward an association with the hemangiopericytoma phenotype and more aggressive behavior in tumors lacking this variant [11]. We studied a large series of patients with SFT/HPC from six tertiary care centers, to determine the best grading scheme for meningeal-based SFT/HPT and characterize the relationship of *NAB2*–*STAT6* fusion status with phenotype and prognosis.

## Materials and methods

This study was reviewed and approved by the Institutional Review Board at all participating institutions.

### Case selection

A cohort of 133 patients with meningeal SFT/HPC (74 males; 59 females) was identified from six tertiary care centers. They ranged in age from 17 to 78 years (median 49.2) at the initial diagnosis. Tumors were pathologically confirmed through review of the first tumor resection (*n* = 97), local recurrence (*n* = 35), or distant metastasis (*n* = 1). Data regarding treatment and clinical follow-up were obtained at the respective institutions based on review of the clinical records.

### Histologic review

**Fig. 1:**
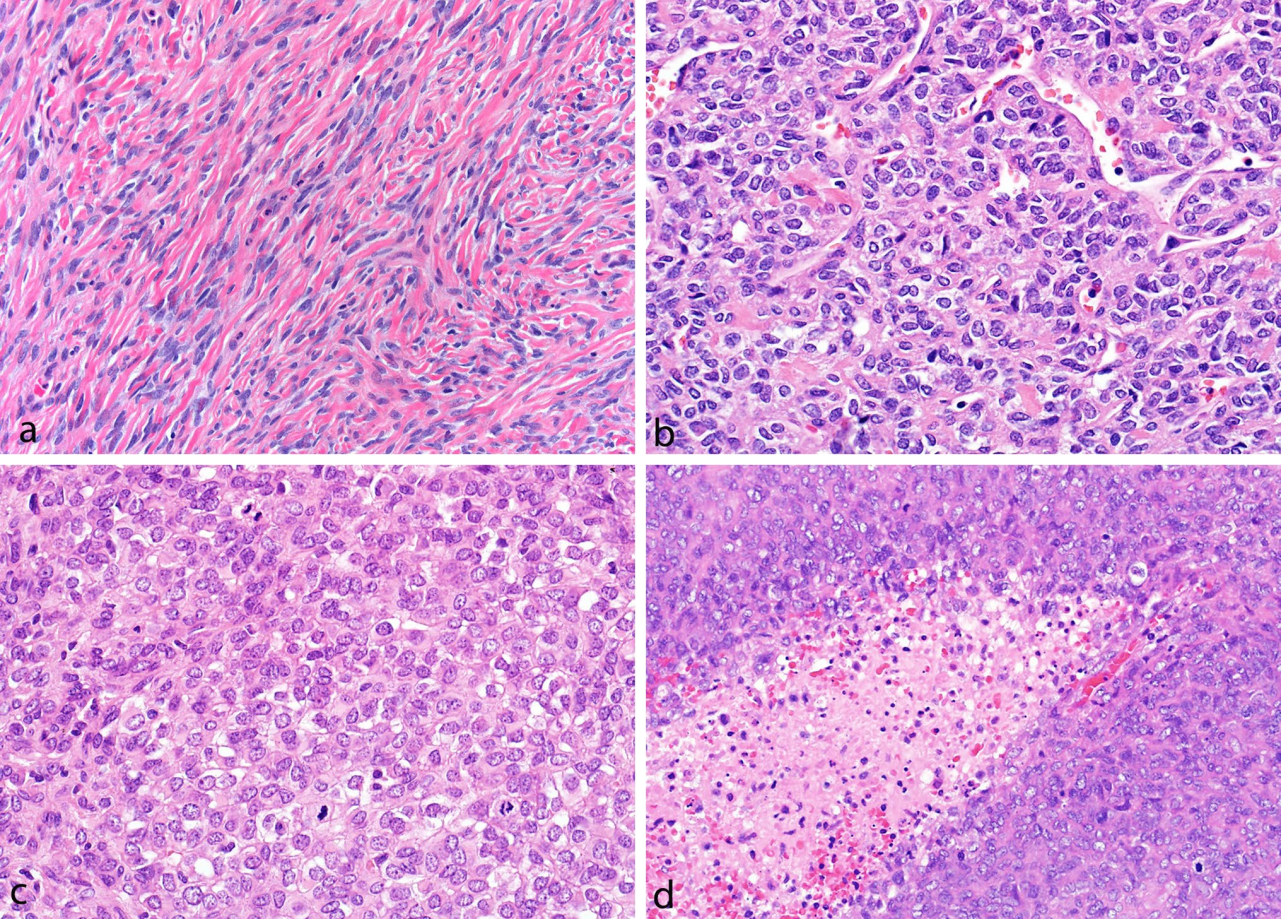
Tumors classified as solitary fibrous tumor contained uniform ovoid-to-slightly spindled-shaped cell deposited in a collagenized background and arranged around branching and hyalinized blood vessels (**a**), while hemangiopericytomas were highly cellular tumors composed of predominantly round cells in a sheet-like pattern (**b**) with less prominent vasculature often showing high mitotic rates (**c**) and necrosis (**d**)

Archived H&E-stained sections were classified phenotypically as ‘SFT’, ‘HPC’, or tumors with intermediate morphological features between HPC and SFT (Fig. 1). SFTs were low-to-moderate cellular tumors composed of spindled to ovoid-shaped cells arranged around branching blood vessels with variable stromal and perivascular hyalinization. HPCs harbored ovoid-to-round cells, often in a sheet-like growth pattern, with high N:C ratios and scant, amphophilic to-clear cytoplasm. Although branching blood vessels were typically present, the vasculature of HPC was frequently less conspicuous than the conventional SFT, and stromal hyalinization was generally absent. Tumors showing the features of both SFT and HPC were considered intermediate. Mitotic rate was assessed by scanning all available H&E slides and counting mitoses (at × 400) in ten consecutive fields in the areas of highest mitotic activity. The presence of necrosis was recorded for all tumors.

### Classification and grading

**Fig. 2:**
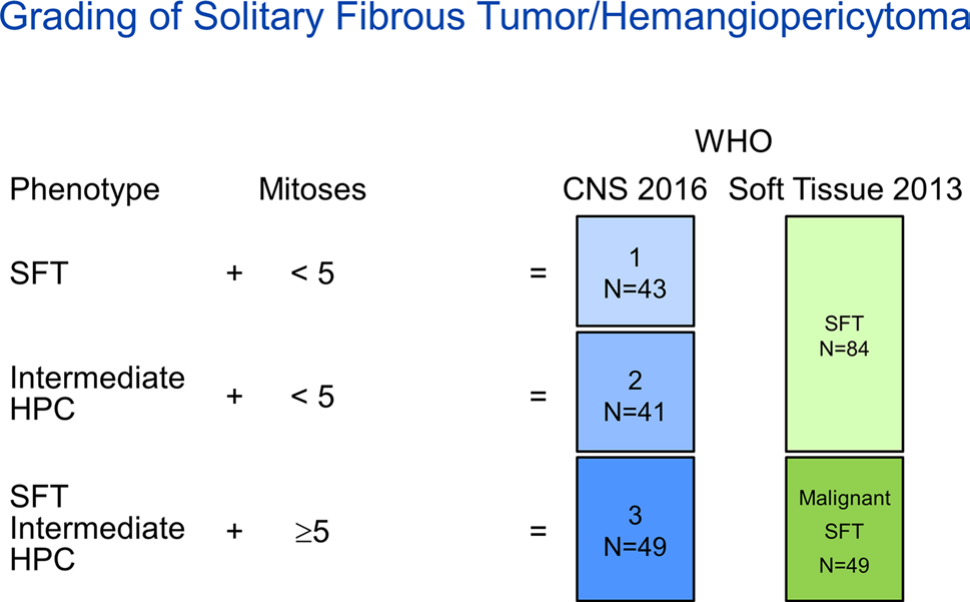
CNS (2016) and Soft Tissue (2013) WHO Classification schemes

Tumors were classified and graded according to the most recent CNS (2016) (CNS-G) and Soft Tissue (2013) (ST-G) WHO classification schemes (Fig. 2). According to the CNS-G, tumors with a classic SFT histopathological phenotype and fewer than five mitoses (× 10 HPF) were considered grade 1; tumors with intermediate or HPC phenotype and fewer than five mitoses (× 10 HPF) were considered grade 2; tumors with five or more mitoses (× 10 HPF) were considered grade 3 irrespective of their histopathological phenotype. According to the ST-G, irrespective of their histopathological phenotype, tumors with fewer than five mitoses (× 10 HPF) were considered SFT and tumors with five or more mitoses (× 10 HPF) were considered malignant SFT.

### Immunohistochemistry

A representative tissue block from each tumor was selected and stained for STAT6 (Santa Cruz Biotechnology, CA, mouse monoclonal antibody, 1:50) and CD34 (Novocastra, liquid mouse monoclonal antibody, 1:100). CD34 immunoreactivity was recorded as negative (< 5%), focal (5–50%), or diffuse (> 50%), while nuclear expression of STAT6 was scored as negative (when nuclear STAT6 expression was not present in tumor cells) or positive (when the cells showed definite STAT6 nuclear expression at least focally in the tumor).

### Molecular studies

RNA extraction was performed by the Mayo Clinic Pathology Research Core using the Qiagen miRNeasy FFPE kit by methods previously described by Wang et al. [20]. RT-PCR of hypoxanthine phosphoribosyltransferase 1 (HPRT1) was used as control for RNA integrity as described previously [3, 11] and samples with degraded RNA were discarded to avoid generation of false-negative cases. In a first step, we screened for the presence of the most common *NAB2*–*STAT6* fusions exon 4–exon 2, exon 4–exon 3, exon 6–exon 16, and exon 6–exon 17 using the well-established single-plex PCR with subsequent agarose gel electrophoresis, identifying *NAB2*–*STAT6* gene fusions in 52 samples. In a next step, samples with good RNA quality but no result in the single-plex PCRs were analyzed by next-generation sequencing using the Archer FusionPlex Sarcoma Kit (ArcherDx, Inc, Boulder, CO, USA) to detect fusions among 26 genes employing the Anchored Multiplex PCR-based enrichment, identifying *NAB2*–*STAT6* gene fusions in an additional 47 samples. Briefly, a total of up to 250 ng RNA were used as starting material. Library preparation was performed according to the manufacturer’s protocol (ArcherDx, Inc) and sequencing was done on a NextSeq550 instrument using NextSeq500/550 High Output v2 kit (150 cycles) (Illumina, Inc., San Diego, CA, USA). The resulting raw data were converted to fastq files and were then processed with the automated Archer Analysis Bioinformatics Platform (Version Archer Analysis 5.1, ArcherDx, Inc).

### Statistical methods

Patient and tumor characteristics were summarized with frequencies and percentages or medians, interquartile ranges (IQR), or ranges, as appropriate. Fisher’s exact tests were used to compare categorical variables between selected groups (e.g., CNS-G, ST-G, and molecular cluster type). Our correlative analysis was based on the time of surgery, which represents when the first tumor was evaluated at the institution. This was the primary tumor resection for 97 patients, resection of a recurrent tumor for 35 patients, and of a metastasis for 1 patient. This approach was made necessary by the fact that, in 36 patients, the primary tumor was not available, and no assumption could be made regarding the grade of the primary tumor. A recent study has shown histological progression of tumor at recurrence in 16% of cases [2]. Recurrence-free survival (RFS) was defined as the time between surgery and the first adverse event (local recurrence or metastasis) following surgery, censoring patients with no adverse event at time of last follow-up. Overall survival (OS) was defined as the time between surgery and death (any cause), censoring those still alive at last follow-up. OS and RFS were estimated using the Kaplan–Meier method, and were summarized at 5, 10, and 20-year post-surgery, along with the median survival. OS and RFS were compared between selected groups with Cox proportional-hazard regression models, using the likelihood ratio test to assess significance. 95% confidence intervals (CI) were also reported for the survival estimates and hazard ratios (HR). We also investigated RFS from time of original diagnosis to the first adverse event in the patient’s history (this also considers the time interval prior to when a recurrent sample was evaluated), as well as OS from time of original diagnosis. For disease-free survival, deaths due to disease were the events, and all non-events (deaths with unknown or other causes, along with those still alive) were censored. All analyses were performed using SAS version 9.4 (SAS Institute Inc, Cary, NC, USA), or R [19]. *p* values less than 0.05 were considered statistically significant.

## Results

Table 1 summarizes the characteristics of the patient cohort.

**Table 1:** Clinicopathologic features

	Total (*N* = 133)^a^	Specimen type at time of surgery		
		Primary (*N* = 97)^a^	Recurrence (*N* = 35)^a^	Metastasis (*N* = 1)^a^
Age at surgery				
Median	54.1	54.1	55.3	41.4
Range	(20.1–87.3)	(20.1–83.0)	(39.0–87.3)	(41.4–41.4)
Age at initial diagnosis				
Median	49.2	52.9	39.3	
Range	(17.5–78.8)	(20.1–78.8)	(17.5–61.1)	
Sex				
Female (%)	59 (44.4)	42 (43.3)	17 (48.6)	0
Male	74 (55.6)	55 (56.7)	18 (51.4)	1
Race/ethnicity				
Caucasian (%)	103 (77.4)	77 (79.4)	26 (74.3)	0
Hispanic/Latino	6 (4.5)	5 (5.2)	1 (2.9)	0
African American	9 (6.8)	7 (7.2)	2 (5.7)	0
Native American	2 (1.5)	0 (0)	2 (5.7)	0
Asian	3 (2.3)	3 (3.1)	0 (0)	0
Pacific Islander	2 (1.5)	1 (1.0)	1 (2.9)	0
Other	2 (1.5)	1 (1.0)	1 (2.9)	0
Unknown	6 (4.5)	3 (3.1)	2 (5.7)	1
Phenotype				
HPC (%)	24 (18.0)	15 (15.5)	9 (25.7)	0
INT	54 (40.6)	39 (40.2)	14 (40.0)	1
SFT	55 (41.4)	43 (44.3)	12 (34.3)	0
Tumor size (cm)				
*N*	96	75	21	0
Median	4.1	4.5	3.8	
Q1, Q3	3.3, 6.0	3.1, 6.5	3.3, 5.0	
Range	(1.3–11.0)	(1.3–11.0)	(1.4–6.5)	
Mitoses (/10 hpf)				
Median	2.0	1.0	3.0	16.0
Q1, Q3	1.0, 6.0	1.0, 6.0	1.0, 8.0	
Range	(0.0–45.0)	(0.0–36.0)	(0.0–45.0)	
Necrosis				
Absent (%)	117 (88.0)	87 (89.7)	29 (82.9)	1
Present	16 (12.0)	10 (10.3)	6 (17.1)	0
CNS-G grade				
1 (%)	43 (32.3)	36 (37.1)	7 (20.0)	0
2	41 (30.8)	28 (28.9)	13 (37.1)	0
3	49 (36.8)	33 (34.0)	15 (42.9)	1
ST-G grade				
Low (%)	84 (63.2)	64 (66.0)	20 (57.1)	0
High	49 (36.8)	33 (34.0)	15 (42.9)	1
Extent of resection				
Gross total resection (%)	63 (56.3)	55 (62.5)	8 (33.3)	0
Subtotal resection	49 (43.8)	33 (37.5)	16 (66.7)	0
CD34				
Negative (< 5) (%)	25 (18.8)	19 (19.6)	6 (17.1)	0
Focal (5–50)	32 (24.1)	21 (21.6)	10 (28.6)	1
Diffuse (> 50)	76 (57.1)	57 (58.8)	19 (54.3)	0
Treatment				
Radiation only (%)	60 (53.1)	50 (58.1)	10 (37.0)	0
Chemotherapy only	1 (0.9)	1 (1.2)	0 (0.0)	0
Radiation + chemotherapy	3 (2.7)	2 (2.3)	1 (3.7)	0
No treatment	49 (43.4)	33 (38.4)	16 (59.3)	0
Molecular cluster				
ex2–3_ex18/ex2_ex1–2/other (%)	10 (7.5)	8 (8.2)	2 (5.7)	0
ex4_ex2–3	29 (21.8)	22 (22.7)	7 (20.0)	0
ex5–7_ex16–17	60 (45.1)	46 (47.4)	14 (40.0)	0
No fusion detected	12 (9.0)	8 (8.2)	3 (8.6)	1
Failed or unavailable	22 (16.5)	13 (13.4)	9 (25.7)	0

^a^Frequencies not summing to column total indicate missing data. For continuous variables, N is provided in cases of missing data

### Clinical outcome

**Fig. 3:**
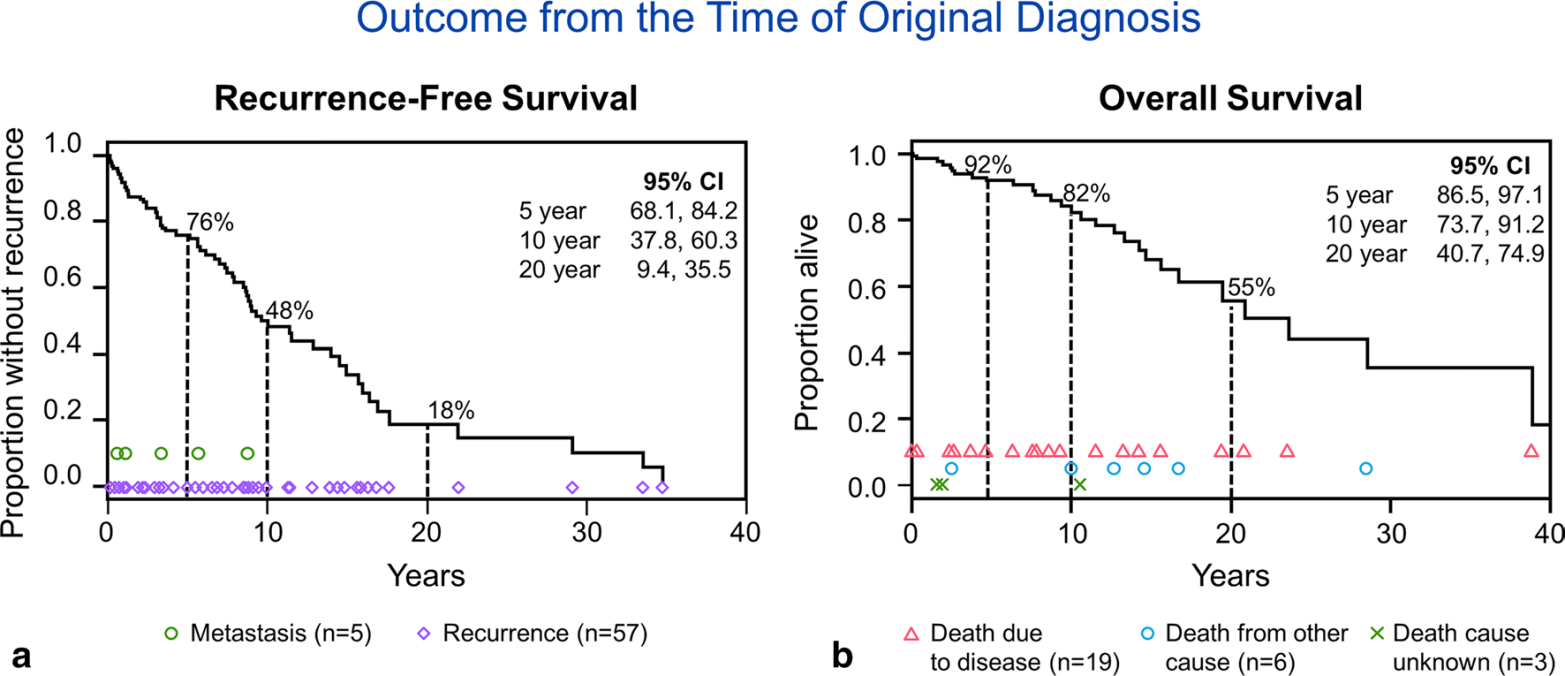
Kaplan–Meier plots showing the median recurrence-free survival (RFS) and overall survival (OS) from the time of the original diagnosis, 9.6 years and 20.9 years, respectively. Note that there was one death due to disease with unknown date of death and that patient is not included in these plots

Follow-up was available in 129 of 133 patients (range 4 days–22.2 years; median 5 years). At last follow-up, 104 patients were alive (47 without disease, 24 with disease, and 33 alive but their disease status was unknown) and 29 patients were deceased (20 of disease, 6 of other causes, and 3 of unknown cause). The median recurrence-free (RFS) and overall survival (OS) times from the original diagnosis were 9.6 years and 20.9 years, respectively (Fig. 3). When RFS and OS were calculated from the time at which the first surgical specimen was available for pathology review (indicated as time of surgery), the median RFS and OS were 11.3 years and 14.7 years, respectively. The differences in RFS and OS with respect to time of diagnosis versus time of surgery are explained by the influence of the patients in which the first available specimen was a recurrence rather than a primary tumor, as illustrated in suppl. Figure 1 and suppl. Figure 2 (Online Resource 1 and Online Resource 2). When considering only cases in which the primary tumor was available for review, the median RFS for primary tumors was 12.9 years (79.0% at 5 years) compared to 7.8 years (67.6% at 5 years) for recurrent cases (*p* = 0.14). The median OS from the time of surgery for cases in which the primary tumor was available for review was 14.7 years (89.1% at 5 years) compared to 9.6 years (70.3% at 5 years) for those in which the recurrence was the first available material (*p* = 0.03). The disease-free survival for all patients at 5 years was 87.2% (95% CI 80.3, 94.1) and at 10 years was 74.2% (95% CI 62.6, 85.7). For patients in which the primary tumor was reviewed, the 5- and 10-year survivals were 91.5% (95% CI 84.9, 98.1) and 85.5% (95% CI 75.2, 95.7), respectively, while the 5- and 10-year survivals for patients in which a recurrence was reviewed was 85.5% (95% CI 75.2, 95.7) and 43.8% (16.3, 71.3), respectively.

Of the entire cohort, 42 patients experienced at least one adverse event after surgery (local recurrence or metastasis): 7 patients had metastases only, 33 had local recurrence only, and 2 patients had both local recurrence and metastases (Table 2). When looking only at the patients from whom the primary tumor was reviewed (*n* = 97), 28 patients experienced either recurrence or metastasis: 4 patients had metastases only, 22 patients had recurrence only, and 2 patients had both recurrence and metastases.

**Table 2:** Recurrence and metastasis since original diagnosis

	*N* = 133 Patients	
	Recurrence	Metastasis
# Of events per patient		
0	74	116
1	36	15
2	13	2
3+	10	0
First event after surgery	34	8
Metastasis since diagnosis, site^a^	28 metastases from 17 patients	
Bone (includes sternum)	7	
Liver	7	
Lung	7	
Brain	2	
Kidney	1	
Pancreas	1	
Other (includes CSF, adnexa, epidural)	3	

^a^17 patients had at least one metastasis (five of these patients’ metastasis was in multiple sites). Two of these patients had two metastases (brain followed by brain; bone followed by epidural)

Among those in the full cohort who experienced an adverse event, the median time from original diagnosis to the first local recurrence was 5.6 years (range 27 days–36.7 years), while the median time to first metastasis was 8.8 years (range 7.1 months–23.4 years). For patients from whom the primary tumor was reviewed, the median time to first recurrence was 3.5 years (range 27 days–14.9 years) with median time to first metastasis of 4.5 years (range 7.1 months–10.3 years).

### Histopathology

By histologic criteria, 55 tumors were classified as SFT phenotype, 24 as HPC phenotype, and the remaining 54 were considered intermediate. The median mitotic rate was 2 (× 10 HPF) (IQR 1–6; range 0–45 mitoses per 10 HPF). Necrosis was identified in 16 cases (12%), while 117 tumors lacked this finding. On univariate analysis, mitotic rate (≥ 5 mitoses × 10 HPF) and necrosis were both significantly associated with recurrence-free survival (RFS, *p* = 0.004, *p* = 0.002, respectively) but not overall survival (OS, *p* = 0.12, *p* = 0.54) (Tables 3, 4). Five- and ten-year RFS were 51.1% and 0%, respectively, for tumors with necrosis and 80.1% and 56% for those without necrosis (Table 3).

**Table 3:** Recurrence-free survival (RFS)

Variable	*N*	Events	Median years (95% CI)^a^	5-Year RFS % (95% CI)^a^	10-Year RFS (95% CI)^a^	Hazard ratio (95% CI)	*p* value
Phenotype							0.87
HPC	24	7	7.8 (5.7–NA)	81.1% (64.2%, 97.9%)	41.7% (8.2%, 75.2%)	(Reference)	
INT	54	18	12.9 (8.8–NA)	78.8% (66.3%, 91.3%)	52.7% (34.6%, 70.8%)	0.88 (0.38, 2.30)	
SFT	55	17	11.3 (6.1–NA)	71.3% (57.4%, 85.3%)	53.1% (34.4%, 71.9%)	1.06 (0.45, 2.74)	
Mitoses							0.004
< 5	84	21	14.9 (8.9–NA)	85.0% (76.4%, 93.7%)	58.7% (42.8%, 74.6%)	(Reference)	
≥ 5	49	21	5.7 (3.0–12.9)	59.6% (43.3%, 76.0%)	33.6% (13.9%, 53.2%)	2.48 (1.34, 4.59)	
Necrosis							0.002
Absent	117	33	12.9 (8.2–NA)	80.1% (71.8%, 88.5%)	55.7% (42.5%, 68.9%)	(Reference)	
Present	16	9	5.6 (1.5–8.8)	51.1% (25.0%, 77.3%)	0.0% (0.0%, 0.0%)	4.04 (1.76, 8.51)	
Tumor size							0.46
0–5 cm	52	12		78.1% (65.3%, 90.9%)	61.6% (41.9%, 81.4%)	–	
≥ 5 cm	44	18	8.8 (5.6–)	71.3% (56.9%, 85.8%)	46.2% (27.4%, 65.1%)	1.31 (0.64, 2.80)	
CNS grade (2016)							0.01
1	43	11	NA	81.9% (68.6%, 95.1%)	61.0% (40.5%, 81.5%)	(Reference)	
2	41	10	14.9 (8.9–NA)	88.2% (77.3%, 99.2%)	58.3% (35.3%, 81.2%)	0.80 (0.33, 1.90)	
3	49	21	5.7 (3.0–12.9)	59.6% (43.3%, 76.0%)	33.6% (13.9%, 53.2%)	2.21 (1.08, 4.78)	
Soft-tissue grade (2013)							0.004
SFT	84	21	14.9 (8.9–NA)	85.0% (76.4%, 93.7%)	58.7% (42.8%, 74.6%)	(Reference)	
Malignant SFT	49	21	5.7 (3.0–12.9)	59.6% (43.3%, 76.0%)	33.6% (13.9%, 53.2%)	2.48 (1.34, 4.59)	
Modified soft-tissue grade							0.0006
SFT	84	21	14.9 (8.9–)	85.0% (76.4%, 93.7%)	58.7% (42.8%, 74.6%)	(Reference)	
Malignant SFT no necrosis	36	12	7.8 (3.3–)	68.1% (49.2%, 87.0%)	46.7% (22.7%, 70.7%)	1.75 (0.83, 3.52)	
Malignant SFT with necrosis	13	9	2.0 (1.2–8.8)	42.7% (14.7%, 70.8%)	Not estimable*	6.03 (2.53, 13.34)	
Resection extent							0.27
Gross total	63	18	12.9 (8.8–NA)	79.1% (67.4%, 90.8%)	60.0% (42.5%, 77.5%)	(Reference)	
Subtotal	49	19	7.8 (5.7–NA)	69.6% (55.7%, 83.5%)	42.6% (23.5%, 61.6%)	1.44 (0.75, 2.77)	
Radiation							0.79
No	50	16	9.2 (7.8–NA)	75.5% (62.1%, 88.8%)	48.9% (27.4%, 70.3%)	(Reference)	
Yes	63	20	12.9 (6.1–NA)	78.5% (67.0%, 89.9%)	50.8% (32.2%, 69.3%)	0.91 (0.47, 1.80)	
Molecular cluster (most common types)							0.80
ex4_ex2–3	29	9	11.3 (3.3–NA)	68.2% (48.6%, 87.8%)	59.7% (36.5%, 82.9%)	(Reference)	
ex5–7_ex16–17	60	22	8.8 (6.4–NA)	74.0% (61.7%, 86.4%)	42.7% (25.6%, 59.9%)	1.03 (0.49, 2.36)	
No fusion detected	12	4	12.9 (1.3–NA)	90.0% (71.4%, 100.0%)	72.0% (37.1%, 100.0%)	0.72 (0.19, 2.26)	
Fusion status							0.52
No fusion detected	12	4	12.9 (1.3–NA)	90.0% (71.4%, 100.0%)	72.0% (37.1%, 100.0%)	(Reference)	
Fusion detected (any type)	99	33	8.9 (6.9–NA)	73.0% (62.9%, 83.0%)	46.5% (32.2%, 60.7%)	1.39 (0.54, 4.74)	

^a^NA indicates that the value was not able to be estimated

**Table 4:** Overall survival (OS)

Variable	*N*	Events	Median years (95% CI)^a^	5-Year OS % (95% CI)^a^	10-Year OS (95% CI)^a^	Hazard ratio (95% CI)	*p* value
Phenotype							0.25
HPC	24	8	12.7 (4.8–16.8)	73.7% (51.0%, 96.3%)	55.3% (19.7%, 90.8%)	(Reference)	
INT	54	13	NA	79.8% (67.8%, 91.8%)	71.8% (56.7%, 87.0%)	0.54 (0.22, 1.38)	
SFT	55	8	14.7 (8.7–)	92.1% (83.6%, 100.0%)	63.4% (37.2%, 89.5%)	0.44 (0.16, 1.19)	
Mitoses							0.12
< 5	84	16	14.7 (10.6–NA)	90.5% (83.3%, 97.8%)	69.7% (53.6%, 85.7%)	(Reference)	
≥ 5	49	13	13.3 (5.3–NA)	71.1% (55.2%, 87.0%)	66.6% (49.5%, 83.8%)	1.80 (0.85, 3.76)	
Necrosis							0.54
Absent	117	26	14.7 (10.6–NA)	85.2% (77.6%, 92.7%)	68.7% (55.9%, 81.6%)	(Reference)	
Present	16	3	NA	72.0% (43.9%, 100.0%)	72.0% (43.9%, 100.0%)	1.49 (0.35, 4.38)	
Tumor size							0.22
0–5 cm	52	9	14.7 (–)	79.4% (65.7%, 93.1%)	75.8% (61.0%, 90.6%)		
≥ 5 cm	44	8	16.8 (10.0–)	95.1% (88.4%, 100.0%)	71.9% (51.0%, 92.7%)	0.53 (0.19, 1.46)	
CNS grade (2016)							0.23
1	43	6	14.7 (8.7–NA)	93.7% (85.3%, 100.0%)	67.1% (40.0%, 94.3%)	(Reference)	
2	41	10	12.7 (9.6–NA)	87.5% (76.0%, 99.0%)	70.7% (51.1%, 90.4%)	1.47 (0.55, 4.34)	
3	49	13	13.3 (5.3–NA)	71.1% (55.2%, 87.0%)	66.6% (49.5%, 83.8%)	2.26 (0.89, 6.45)	
Soft-tissue grade (2013)							0.12
SFT	84	16	14.7 (10.6–NA)	90.5% (83.3%, 97.8%)	69.7% (53.6%, 85.7%)	(Reference)	
Malignant SFT	49	13	13.3 (5.3–NA)	71.1% (55.2%, 87.0%)	66.6% (49.5%, 83.8%)	1.80 (0.85, 3.76)	
Modified soft-tissue grade							0.29
SFT	84	16	14.7 (10.6–)	90.5% (83.3%, 97.8%)	69.7% (53.6%, 85.7%)	(Reference)	
Malignant SFT no necrosis	36	10	13.3 (4.8–)	72.7% (54.5%, 90.9%)	66.7% (46.5%, 86.8%)	1.73 (0.75, 3.79)	
Malignant SFT with necrosis	13	3		67.3% (35.6%, 99.0%)	67.3% (35.6%, 99.0%)	2.10 (0.48, 6.43)	
Resection extent							0.05
Gross total	63	8	16.8 (12.7–NA)	93.0% (85.4%, 100.0%)	77.2% (59.3%, 95.1%)	(Reference)	
Subtotal	49	14	13.3 (7.5–NA)	76.8% (63.3%, 90.4%)	62.8% (44.3%, 81.3%)	2.33 (0.996, 5.86)	
Radiation							0.30
No	50	11	12.7 (10.0–NA)	87.2% (76.6%, 97.8%)	63.8% (41.1%, 86.6%)	(Reference)	
Yes	63	10	16.8 (13.3–NA)	86.5% (76.3%, 96.6%)	76.0% (59.6%, 92.3%)	0.64 (0.26, 1.52)	
Molecular cluster (most common types)							0.29
ex4_ex2–3	29	6	12.7 (8.7–NA)	81.5% (65.0%, 97.9%)	65.2% (33.7%, 96.6%)	(Reference)	
ex5–7_ex16–17	60	11	NA	87.0% (77.1%, 96.9%)	74.9% (59.6%, 90.2%)	0.80 (0.30, 2.32)	
No fusion detected	12	7	10.0 (1.6–NA)	61.9% (32.1%, 91.7%)	41.3% (10.6%, 71.9%)	1.79 (0.58, 5.68)	
Fusion status							0.13
No fusion detected	12	7	10.0 (1.6–NA)	61.9% (32.1%, 91.7%)	41.3% (10.6%, 71.9%)	(Reference)	
Fusion detected (any type)	99	18	14.3 (12.7–NA)	84.9% (76.6%, 93.2%)	73.3% (60.5%, 86.2%)	0.48 (0.20, 1.25)	

^a^NA indicates that the value was not able to be estimated

### Tumor size

Tumor size was available in 96 patients ranging from 1.3 to 11.0 cm (Table 1). Tumor size was not significantly associated with RFS or OS when considered continuously or categorically (tumor size < 5 cm vs tumor size ≥ 5 cm) (Tables 3, 4).

### Immunohistochemistry

CD34 was recorded as diffuse in the majority of cases (*n* = 76; 57%), while 32 cases (24%) were focally positive and 25 were negative (19%). STAT6 immunostain showed nuclear expression in 132 (99%) cases. The single case lacking nuclear expression of STAT6 was a T11 spinal mass from a 40-year-old woman that recurred multiple times. STAT6 immunochemistry was performed on two separate specimens from this patient and repeated at two different centers (Mayo Clinic and the originating institution), and all samples tested were negative. Both specimens of this tumor were tested by PCR and showed an *NAB2* exon 6–*STAT6* exon 16 fusion.

### Grading

Tumors were classified as grade 1 (*n* = 43), 2 (*n* = 41), or 3 (*n* = 49) using the CNS-G, and SFT (*n* = 84) or malignant SFT (*n* = 49) using the ST-G scheme (Fig. 2). When comparing the extent of CD34 immunostaining among those with at least 5% staining, tumors with SFT phenotype were more likely to show diffuse CD34 immunoreactivity (88%) as compared to tumors with HPC and INT phenotype (35% and 63%, respectively), *p* = 0.0001. Furthermore, CNS-G 1 tumors were more likely to show diffuse CD34 immunoreactivity (85%) as compared to CNS-G 2 (56%) or 3 (68%), *p* = 0.03.

**Fig. 4:**
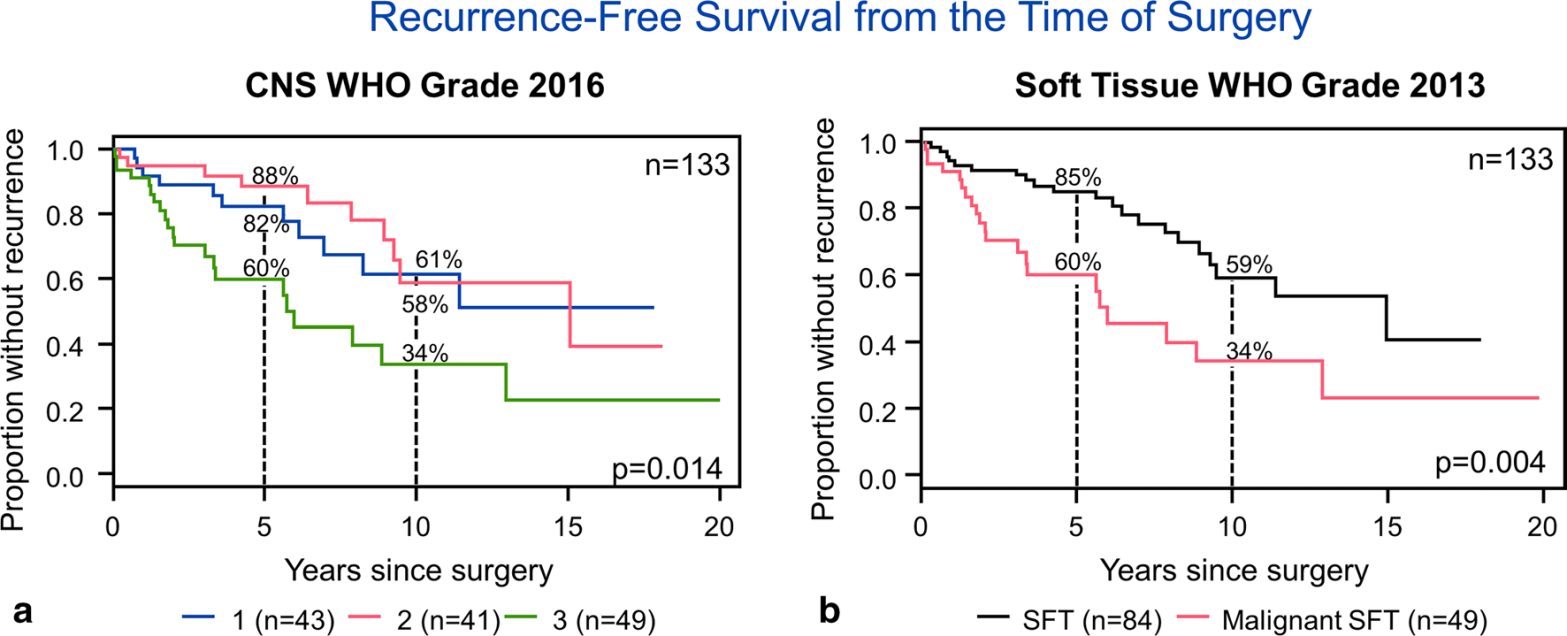
Univariate analysis shows that both the CNS-G (**a**) and ST-G (**b**) are significantly associated with recurrence-free survival (Table 3, *p* = 0.01, 0.004, respectively) but not overall survival (*p* = 0.23, *p* = 0.12, respectively)

**Fig. 5:**
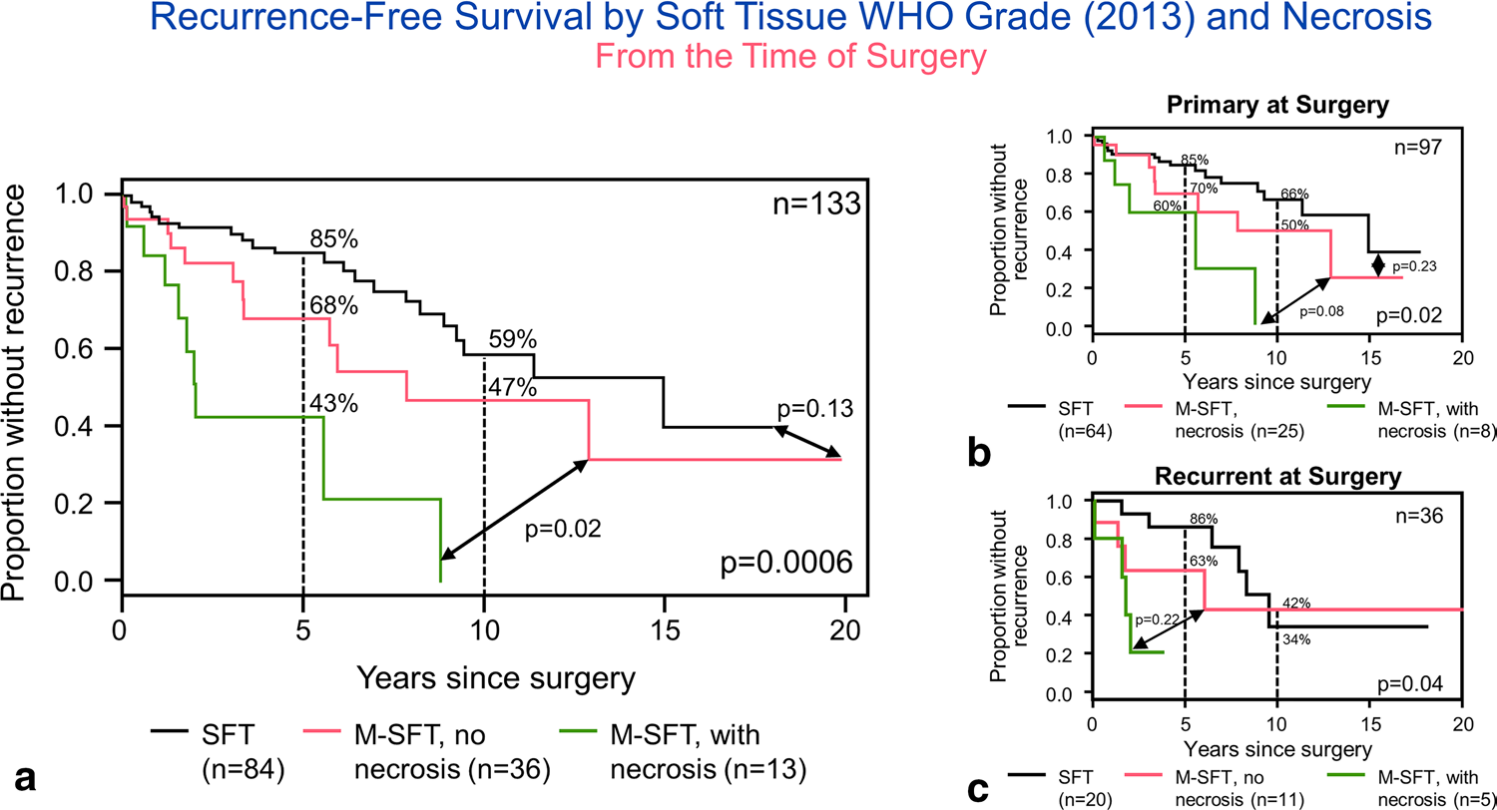
Modified soft-tissue grading scheme showed strong association with recurrence-free survival (*p* = 0.0006) and remained significant (*p* = 0.02) when considering only the primary tumors and only recurrent tumors (*p* = 0.04) (**a**–**c**)

On univariate analysis, both the CNS-G and ST-G were significantly associated with RFS (Table 3, *p* = 0.014, 0.004, respectively) (Fig. 4a, b) but not overall survival (*p* = 0.23, *p* = 0.12, respectively). Five-year RFS rates were, respectively, 82, 88, and 60%, while 10-year rates were 61, 58, and 34% for CNS grades 1, 2, and 3. They were 85 and 60 at 5 years, and 59 and 34% at 10 years for SFT and malignant SFT, respectively. When considering only the primary tumors, ST-G remained significant (*p* = 0.03), while the CNS-G did not (*p* = 0.07). When evaluating only the recurrent cases, the ST-G approached significance (*p* = 0.05), while the CNS-G did not (*p* = 0.15). Since the ST-G scheme appeared to be a simpler way to stratify these tumors, and necrosis was associated with decreased RFS on univariate analysis, we attempted to incorporate necrosis into the ST-G and stratify tumors into three tiers: (1) tumors with < 5 mitoses/10 HPF, (2) tumors with ≥ 5 mitoses/10 HPF without necrosis, and (3) tumors with ≥ 5 mitoses/10 HPF with necrosis. This modified soft-tissue grading scheme showed a strong association with recurrence-free survival (*p* = 0.0006) and remained significant (*p* = 0.02) when considering only the primary tumors and only recurrent tumors (*p* = 0.04) (Fig. 5a–c). Among 84 tumors with < 5 mitoses per 10 HPF, necrosis was present only in 3 (3.6%). All three tumors had a hemangiopericytomatous-like phenotype. In two patients, necrosis was present at the time of primary surgery. One of these patients had GTR and one STR. Both received radiation therapy. They were both alive without recurrence, respectively, at 67 and 38 months. In the third patient, we reviewed a recurrent sample, 16 years and 3 months from the original diagnosis. Only 1 month postoperative follow-up was available in this patient.

### *NAB2–STAT6* fusion

**Fig. 6:**
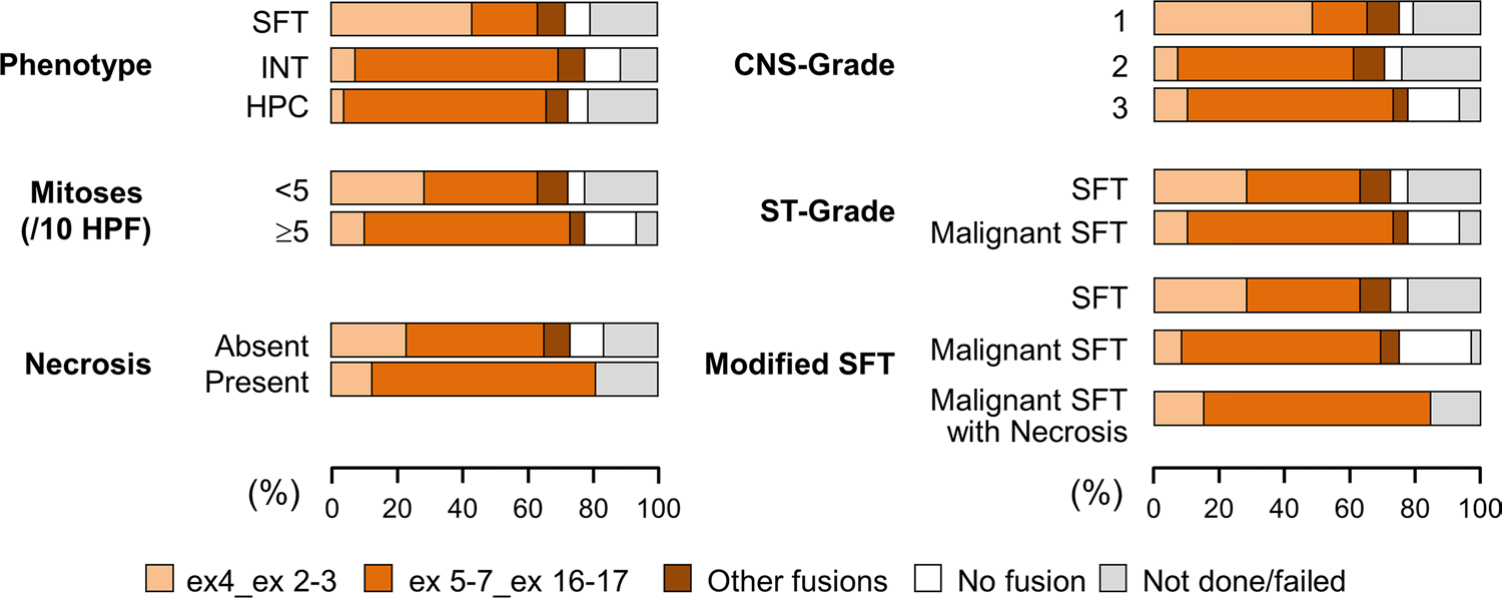
This graph highlights the relationship between the four *NAB2*–*STAT6* fusion groups, and phenotype, mitoses, necrosis, CNS-G, ST-G, and modified ST-G

Molecular analysis was successful in 111 (of 127; 87%) cases which could be tested, and *NAB2*–*STAT6* fusion was detected in 99 (of 111; 89%) successfully tested tumors: *NAB2* exon 5–7–*STAT6* exon 16–17 (*n* = 60), *NAB2* exon 4–*STAT6* exon 2–3 (*n* = 29), *NAB2* exon 2–*STAT6* exon 1–2 (*n* = 4), *NAB2* exon 2–3–*STAT6* exon 18 (*n* = 4), *NAB2* exon 2–*STAT6* exon 5 (*n* = 1), and *NAB2* exon 7–*STAT6* exon 1 (*n* = 1). The remaining 12 cases lacked an identifiable *NAB2*–*STAT6* fusion. Fusion variants were grouped according to their frequency and predicted protein domain inclusion into four groups. The four groups based on fusion variant included: (1) *NAB2* exon 4–*STAT6* exon 2–3 (*n* = 29), (2) *NAB2* exon 5–7–*STAT6* exon 16–17 (*n* = 60), (3) other *NAB2*–*STAT6* fusions (*n* = 10), and (4) no detectable fusion (*n* = 12). Figure 6 illustrates the relationship between the four *NAB2*–*STAT6* fusion groups, and phenotype, mitoses, necrosis, CNS-G, ST-G, and modified ST-G.

*Fusion cluster and phenotype* When comparing fusion cluster with phenotype, the majority of *NAB2* exon 4–*STAT6* exon 2–3 fusions were seen among tumors with an SFT phenotype (*n* = 24 of 29, 83%), with four cases showing intermediate phenotype and one with HPC phenotype. The tumors which harbored *NAB2* exon 5–7–*STAT6* exon 16–17 were more likely to have an intermediate or HPC phenotype (*n* = 49 of 60 and 82%; *n* = 34, and *n* = 15, respectively) (*p* < 0.0001), the remaining 11 (18%) showing an SFT phenotype. Among the ten with other fusions, half (5) were SFT, four intermediate, and one HPC. Among the 12 with no fusion detected, 4 were SFT, 6 intermediate, and 2 HPC.

*Fusion cluster and mitotic rate* Tumors with *NAB2* exon 4–*STAT6* exon 2–3 or “other fusions” were more likely to have mitotic rates < 5/10 HPF, whereas tumors with *NAB2*–*STAT6* 5–7–*STAT6* exon 16–17 and without a detectable fusion were more likely to have mitotic rates ≥ 5/10 HPF (*p* = 0.002).

*Fusion cluster and necrosis* There was no significant association between fusion type or status (absence or presence of a detected fusion) and necrosis (*p* = 0.16; *p* = 0.35, respectively).

*Fusion cluster and CNS*-*G* There was a statistically significant association between type of fusion and CNS-G (*p* < 0.0001). The 29 tumors with *NAB2* exon 4–*STAT6* exon 2–3 were more frequently grade 1 tumors (*n* = 21) than grade 2 or 3 (*n* = 3, *n* = 5, respectively). The 60 tumors with *NAB2* exon 5–7–*STAT6* exon 16–17 fusion were more frequently grade 2 and 3 tumors (*n* = 22, *n* = 31, respectively).

*Fusion cluster and ST*-*G* There was a statistically significant association between type of fusion and ST-G (*p* = 0.002). The 29 tumors harboring *NAB2* exon 4–*STAT6* exon 2–3 or the 10 with “other fusions” were more likely to be solitary fibrous tumors (*n* = 24, *n* = 8) compared to malignant solitary fibrous tumors (*n* = 5, *n* = 2). The 60 tumors with *NAB2* exon 5–7–*STAT6* exon 16–17 were malignant SFT in 31 (52%) and SFT in 29 (48%). The majority of tumors without a detectable fusion were malignant SFT (*n* = 8) compared to SFT (*n* = 4).

*Fusion cluster and outcome NAB2*–*STAT6* fusion type was not associated with RFS (*p* = 0.80) or OS (*p* = 0.29). Furthermore, when comparing tumors with and without identifiable *NAB2*–*STAT6* fusions (dichotomously), there was no significant difference in recurrence-free survival (*p* = 0.52) or overall survival (*p* = 0.13).

### Treatment data

Sixty-three patients (56.3%) underwent gross total resection, while the tumor was subtotally resected in 49 patients (43.8%). In the remaining 21 patients, the extent of surgical resection was unknown. Sixty patients (53.1%) received only adjuvant radiation therapy, a single patient (1%) was treated with chemotherapy alone, 3 patients (2.7%) underwent both adjuvant radiation and chemotherapy, and the remaining 49 patients (43.4%) were treated with surgery alone. Neither extent of resection nor adjuvant radiation therapy was significantly associated with outcome (overall survival: *p* = 0.051, 0.30; recurrence-free survival: *p* = 0.27, 0.79, respectively).

## Discussion

Our study confirms the natural history of meningeal-based SFT/HPC, showing high rates of local recurrence and distant metastasis. Furthermore, patients in our cohort demonstrate a tendency for late adverse events, a subset developing their first recurrences/metastasis over 3 decades after the initial diagnosis. Similarly, patients continue to die of disease almost 40 years after the initial diagnosis. In a subset of our cases, only material from recurrence (not the primary tumor) was available for review. As these patients are at greater risk for adverse events, as shown by our data, these cases influence the OS and RFS of the entire cohort. However, trends for overall survival and RFS of both primary and recurrent cases remain similar.

On univariate analysis, mitotic rate and necrosis were each significantly associated with RFS but not OS. Since the CNS-G and ST-G schemes take mitotic rate into account, it is not surprising that both appear to show association with RFS. However, there does not appear to be a significant difference between grade 1 and grade 2 according to CNS-G, and the ST-G scheme, based solely on mitotic rate, appears to stratify tumors in a simpler and more efficient fashion. Furthermore, the ST-G maintains statistical significance when looking only at primary cases. A recent risk stratification model proposed by Demicco et al. for non-meningeal tumors incorporates mitotic rate and necrosis, along with patient age and tumor size, and appears to show prognostic significance in determining propensity for metastatic disease [8]. The class sizes used in soft tissue with cut-offs of 5, 10, and 15 cm would not be easily applicable to intracranial tumors, which grow in a confined space. However, necrosis was associated with decreased RFS on univariate analysis in our series. Furthermore, incorporating necrosis into ST-G, i.e., classifying tumors based on mitotic rate and necrosis, seems to improve the outcome models when compared to mitotic rate alone. In their recent work from 2018, Macagno et al. sought to validate an updated version of the Marseille Grading Scheme which segregated 132 tumors into three groups based on mitotic activity (≥ 5 mitotic figures/10 HPF) and necrosis [4, 15]. On univariate analysis, they found that extent of surgery, radiotherapy/chemotherapy, and mitotic rate were significant in predicting RFS, while radiation, mitotic rate, and necrosis were significant for disease-specific survival [15]. Extent of surgery and mitotic rate remained significant prognostic factors for RFS with multivariate analysis, while necrosis and radiotherapy were significant for disease-specific survival [15]. Our data parallel these findings, confirming that a grading scheme incorporating mitotic rate and necrosis best stratifies SFT/HPC, and the combination of high mitotic activity and necrosis portends poor prognosis for this entity. If these findings are corroborated by additional studies, this grading scheme could be utilized to guide treatment decisions regarding adjuvant therapy (e.g., radiotherapy) after surgery. Of note is the fact that, once necrosis is added to the ST-G model, the RFS curve of SFT does not appear to be significantly different from that of malignant SFT without necrosis, which, however, remains distinctly separate and inferior. Concern could be raised about applying the term malignant SFT as per the 2013 WHO classification for soft-tissue tumors to these tumors. Larger studies and longer follow-up are needed to answer this question.

In 2014, Barthelmess et al. examined a series of non-meningeal SFT/HPC and showed that tumors harboring *NAB2* exon 4–*STAT6* exon 2/3 fusions had lower recurrence rates than those with *NAB2* exon 6–*STAT6* exon 16/17 or other fusions [3]. Similarly, our subsequent work with meningeal-based lesions suggested a trend toward more aggressive behavior in tumors lacking *NAB2* exon 4–*STAT6* exon 3. Even though additional studies of dural- and non-dural-based solitary fibrous tumors have shown consistent association between mitotic rate and fusion variant, with tumors harboring *NAB2* exon 4–*STAT6* exon 2/3 fusions consistently exhibiting lower mitotic rates than those with *NAB2* exon 6–*STAT6* exon 16/17, no further relationship between fusion variant and disease-free survival has been reported [1, 3, 6, 12, 18, 21]. Our current study that focused specifically on a large cohort of meningeal-based tumors from multiple large tertiary care centers provides further support that fusion status fails to associate with RFS or overall survival.

Interestingly, several studies, including our earlier work, have suggested that fusion type appears to be associated with phenotype. The *NAB2* exon 4–*STAT6* exon 2–3 variant seems to be most often found in tumors resembling the conventional ‘solitary fibrous tumor,’ while tumors with *NAB2* exon 6–*STAT6* exon 16–17 exhibit morphologic features consistent with what was previously considered ‘hemangiopericytoma’ [1, 3, 11]. Our current findings are concordant with these prior reports and suggest that tumor phenotype is related to fusion variant.

The sensitivity of STAT6 for the diagnosis of solitary fibrous tumors has been reported to range from 86 to 98% in large series at both CNS and soft-tissue sites [7, 9, 13, 17]. We identified only a single case of SFT/HPC which harbored an *NAB2* exon 6–*STAT6* exon 16 fusion but lacked nuclear STAT6 immunoreactivity. The tissue blocks from this patient were from 1997 and 1998, and it is possible that the age of the blocks precluded accurate immunohistochemical analysis. We also considered the possibility that the epitope recognized by our antibody may be lost in this specific fusion. However, the fusion in this case is the most frequently recognized, and, consequently, we would not expect loss of STAT6 staining based on fusion type. Regardless, the absence of STAT6 immunostaining in a tumor with morphologic features consistent with SFT/HPC should lead to the consideration of molecular testing to assess for the presence of *NAB2*–*STAT6* fusion.

In conclusion, meningeal SFT/HPC is an aggressive tumor with propensity for high rates of recurrence and metastasis, which sometimes occur decades after the initial diagnosis. A grading scheme incorporating mitotic rate and necrosis seems to stratify this family of tumors most accurately. Although fusion type does not appear to relate to outcome in dural-based lesions, it does seem to be associated with tumor phenotype.

## Electronic supplementary material

Below is the link to the electronic supplementary material.

Suppl. figure 1 (Online Resource 1). The influence on RFS with respect to time of diagnosis versus time of surgery of patients in which the first available material was a recurrence rather than a primary tumor is shown here (TIFF 923 kb)

Suppl. figure 2 (Online Resource 2). The influence on OS with respect to time of diagnosis versus time of surgery of patients in which the first available material was a recurrence rather than a primary tumor is shown here (TIFF 976 kb)
