# Supplementary figures and images for: The impact of histopathology and NAB2–STAT6 fusion subtype in classification and grading of meningeal solitary fibrous tumor/hemangiopericytoma
Source: Acta Neuropathol. 2018 Dec 24;137(2):307–19. doi: 10.1007/s00401-018-1952-6 (PMC6513906; doi:10.1007/s00401-018-1952-6)

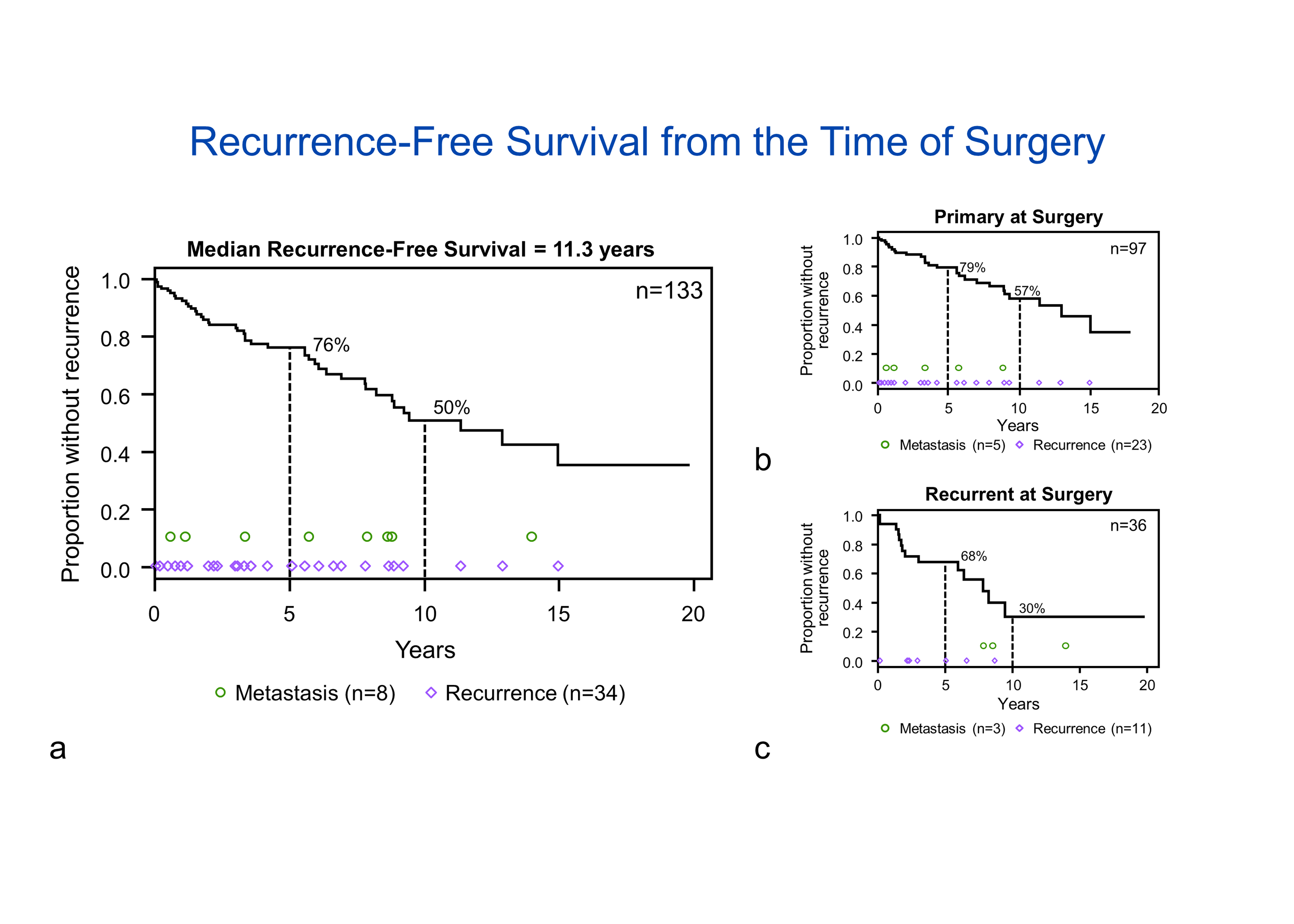

Supplement: Supplementary file 1 — Suppl. figure 1 (Online Resource 1). The influence on RFS with respect to time of diagnosis versus time of surgery of patients in which the first available material was a recurrence rather than a primary tumor is shown here (TIFF 923 kb) [file 401_2018_1952_MOESM1_ESM.tif]

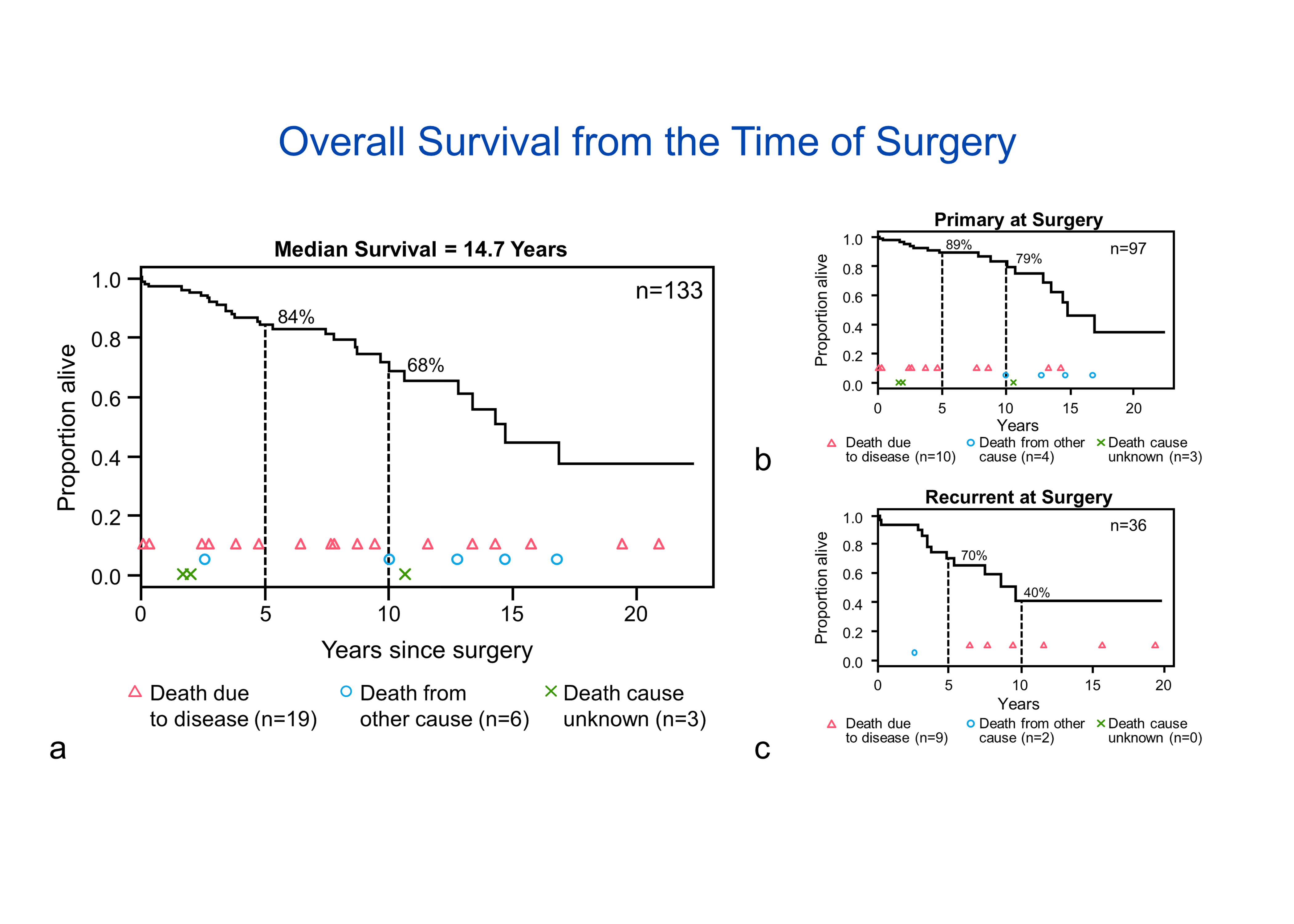

Supplement: Supplementary file 2 — Suppl. figure 2 (Online Resource 2). The influence on OS with respect to time of diagnosis versus time of surgery of patients in which the first available material was a recurrence rather than a primary tumor is shown here (TIFF 976 kb) [file 401_2018_1952_MOESM2_ESM.tif]
